# Factors Influencing Self-Management in Chinese Adults with Type 2 Diabetes: A Systematic Review and Meta-Analysis

**DOI:** 10.3390/ijerph120911304

**Published:** 2015-09-10

**Authors:** Xiaoping Luo, Tingting Liu, Xiaojing Yuan, Song Ge, Jing Yang, Changwei Li, Wenjie Sun

**Affiliations:** 1 Anesthesia Department of Zhongshan People’s Hospital, Zhongshan 528403, China; E-Mail: rober81@163.com; 2 Nell Hodgson Woodruff School of Nursing, Emory University, Atlanta, GA 30322, USA; E-Mail: tingting.liu@emory.edu; 3 Department of Epidemiology, School of Public Health and Tropical Medicine, Tulane University, New Orleans, LA 70112, USA; E-Mails: xyuan3@tulane.edu (X.Y.); cli8@tulane.edu (C.L.); 4 School of Nursing, Johns Hopkins University, Baltimore, MD 21205, USA; E-Mail: sge3@jhu.edu; 5 School of Nursing, Chengdu University of Traditional Chinese Medicine, Chengdu 611137, China; E-Mail: cindyang@foxmail.com; 6 School of Food Science, Guangdong Pharmaceutical University, Zhongshan 528458, China

**Keywords:** Chinese adults, diabetes self-management, type 2 diabetes, systematic review

## Abstract

Diabetes is a major public health problem in China. Diabetes self-management is critical for patients to achieved better health outcomes, however, previous studies have shown suboptimal diabetes self-management performance. We conducted a systematic review and meta-analysis to identify factors associated with diabetes self-management in Chinese adults. The results showed that confrontation, resignation, overall health beliefs, perceived susceptibility, perceived barriers, and self-efficacy were factors associated with overall diabetes self-management performance and six aspects of diabetes self-management behaviors. There is some limited evidence to suggest that provider-patient communication, married individuals, higher educational level, and higher household income level may also be linked to better diabetes self-management practice. Having healthcare insurance and utilizing chronic illness resources generally appeared to have a favorable effect on diabetes self-management performance. In addition, there were a number of factors for which the evidence is too limited to be able to ascertain its strength of association with diabetes self-management practice. The findings of this review suggest that diabetes self-management behaviors are affected by a wide range of personal and environmental factors, which allow health care providers to develop theory-based strategies to improve diabetes-self-management behaviors in this population.

## 1. Introduction

Diabetes is a major public health problem worldwide and it is increasing by epidemic proportions. Globally, the total number of people living with diabetes is projected to rise from 382 million cases in 2013 to 592 million cases by 2035, with over 80% of cases living in low- and middle-income countries [1]. In China alone, about 114 million (11.6%) adults had diabetes in 2010, a two-fold increase over the past decade [2]. The disease burden resulting from diabetes has translated into a substantial economic toll. The estimated annual direct cost to the Chinese national health service for treating diabetes was ¥173.4 billion (about $28.5 billion), which accounted for roughly 13% nation’s total medical expenditures in 2010 [3]. Facilitating diabetes care is thus important for nurses and other providers of primary care.

### Background

Type 2 diabetes (T2D) accounts for approximately 90% to 95% of all diagnosed cases of diabetes [4]. It is estimated that in China 102.5 to 108.2 million individuals have T2D. Individuals with T2D perform about 95% of their own care [5]. Diabetes self-management (DSM) is an essential element of diabetes care, and refers to daily behaviors that individuals perform to manage their T2D such as self-monitoring blood glucose (SMBG), diet and physical activity [6]. DSM is complex, requires major lifestyle changes and behavioral tasks that are incorporated into an individual’s daily routine and high levels of adherence for effective management and halting disease progression [7]. Convincing evidence has shown that improving DSM was important to achieve better health outcomes [8], including better glycemic control [9], improved quality of life [10], and reduced incidence of complications.

Several studies have consistently shown that DSM practice is suboptimal among Chinese adults with T2D. Poor adherence to DSM behaviors, such as SMBG and foot care, has been documented in this population [11,12,13]. The reason for poor adherence to DSM among Chinese adults is not well known, and this suggests a critical need to identify factors associated with DSM behaviors and develop interventions to target such factors. Although a substantial progress has been made in identifying these factors in recent years, no prior literature reviews have been conducted to synthesize these factors and relative importance of these factors in China. Therefore, the purpose of this study is to synthesize findings of factors associated with DSM among Chinese adults with T2D.

## 2. Methods

### 2.1. Design

The systematic review was performed according to the Centers for Reviews and Dissemination’s guidance for undertaking reviews in health care (hereafter referred to as ‘guidance’) [14] on quantitative studies. The research question is framed in terms of the PICOS elements, which is described in Supplementary Table S1. The inclusion criteria of this review were: (a) research was primarily designed to examine the association between related factors and DSM behaviors; (b) Chinese adults aged 18 years or older with T2D; (c) articles published in either Chinese or English; (d) case-control, cross-sectional, or cohort studies; (e) the research was conducted in Mainland China. Exclusion criteria were: (a) case series and case reports; (b) previous research syntheses; (c) studies recruited adults with type 1 diabetes or both type 1 diabetes and T2D.

Eligible studies were retrieved from four English language databases: PubMed (1966–2014), CINAHL (1982–2014), Web of Science (1976–2014), and EMBASE (1974–2014), and three major Chinese databases: Wanfang Data (1982–2014), Chongqing VIP (1989–2014), and China National Knowledge Infrastructure (1994–2014). The search terms used in PubMed are shown in Supplementary Table S2. Additional eligible articles were identified by hand searching the reference lists of retrieved studies. Unpublished master’s theses and doctoral dissertations written in English or Chinese were also searched. Process for study selection was conducted in two stages: an initial screening of titles and abstracts against the predetermined inclusion/exclusion criteria, followed by a second screening of the full text of the research reports identified as probably relevant in the initial screening. Both stages were carried out independently by two authors, and disagreements resolved by discussion with other authors determined final eligibility of the included articles.

### 2.2. Quality Appraisal

The guidance recommended the use of quality assessment checklist system for observational studies developed by the U.S. Agency for Healthcare Research and Quality [15]. Study questions, study population, comparability of subjects, exposure, outcome measurement, statistical analysis, and funding/sponsorship were considered as key domains to rate quality of observational studies [15]. The quality assessment checklist used in this review was adapted from previously published assessment checklists [16,17] to fully address these domains. Each item was coded as “Y” (yes), “N” (no), “P” (partial), “U” (unclear), or “N/A” (not applicable). Two authors independently performed the quality assessment, with a third author serving as arbiter of conflicts. The quality appraisal results of included studies are presented in Supplementary Table S3.

### 2.3. Data Extraction

Two authors independently performed the data extraction. Any disagreement between the two authors was resolved by discussion with other authors until a consensus was made. Authors of primary studies were contacted to provide missing information. The following data collected for this review were adapted from the guidance [14]: author, year, purpose of study, follow-up period, study design, sample size, age, gender, socioeconomics, name of the factors, beta/or correlation coefficient on DSM, and any further relevant information was described as ‘other’.

### 2.4. Statistical Analysis

The report of the review had a narrative summary and was developed according to the Preferred Reporting Items for Systematic Reviews and Meta-analyses criteria [18]. For factors where results were reported in a similar fashion and had consistent definitions across studies, a meta-analysis was performed to obtain the associations between these factors and DSM behaviors. The measure of association from included articles was β coefficients, correlation coefficients, or odds ratios (OR). Standard error and/or 95% confidence interval (CI) of β, correlation coefficients, or OR were also collected. When meta-analysis is not possible owing to heterogeneity, or for factors which were reported in a single study, a textual summary of findings was presented.

In order to achieve better generalizability of study results, a random effects model was used. The Cochran Q statistic and I^2^ statistic were used to test for heterogeneity. Studies were weighted by the inverse-variance. Because the sampling distribution for r was skewed, Fisher’s z-transformation was performed to achieve a normal distribution. Correlation coefficients were transformed into Fisher’s z scores for generating overall estimates and were transformed to its origin format for interpreting results. Beta coefficients were pooled only when the same instrument to measured DSM was used. Pooled β coefficients or Fisher’s z scores were calculated using inverse-variance weighted DerSimonian and Laird procedure for random effects meta-analysis. The Begger’s and Egger’s test was performed to assess publication bias. The Duval and Tweedie trim and fill method was used to see if publication bias influenced the results. The significant level of the entire analysis was chosen at α = 0.05. All analyses were performed in STATA version 12.0 (StataCorp LP, College Station, TX, USA).

## 3. Results

### 3.1. Search Outcomes

The initial search strategy identified 51 articles across the four English databases. The number of hits on the databases specified above was as follows (in blankets): PubMed (19); CINAHL (1); Web of Science (16); and EMBASE (15). After title and abstract review of the four English databases, six articles were considered relevant and were included. A total of 45 articles were excluded, among which five articles were duplicates across databases, 32 studies were not designed primarily to examine which factors were related to DSM behaviors, and the remaining seven articles were carried out in places other than Mainland China.

In addition, three articles were identified as relevant by a manual search. The nine articles were then downloaded for full-text review. After careful content reading, two articles were considered as multiple reports of the same study [19,20]. The two articles were treated as a single study but reference made to both publications. One article did not fulfill the inclusion criteria and therefore was excluded [21]. In sum, seven English articles were included. Search of the ProQuest Dissertations and Theses database found five doctoral dissertations. After title and abstract review, only one dissertation was considered relevant and included in the review [22]. However, the dissertation was published in 2008 [23] and was therefore included among the seven articles, therefore, the dissertation was excluded as a duplicate.

The initial search strategy identified 629 articles across the three Chinese databases. After title and abstract review of the three Chinese databases, 26 articles were considered relevant. A total of 603 articles were excluded, among which 28 articles were duplicates across databases, and 575 studies did not meet the inclusion criteria. Nine articles were identified as relevant by hand search. Thirty-five articles were then downloaded for full-text review. After careful content reading, these articles were considered relevant. Search of the China National Knowledge Infrastructure found 11 doctoral dissertations or master’s theses. After title and abstract review, five master theses were considered relevant and included in the review. In sum, a total of 40 Chinese articles were included in the review.

**Figure 1:**
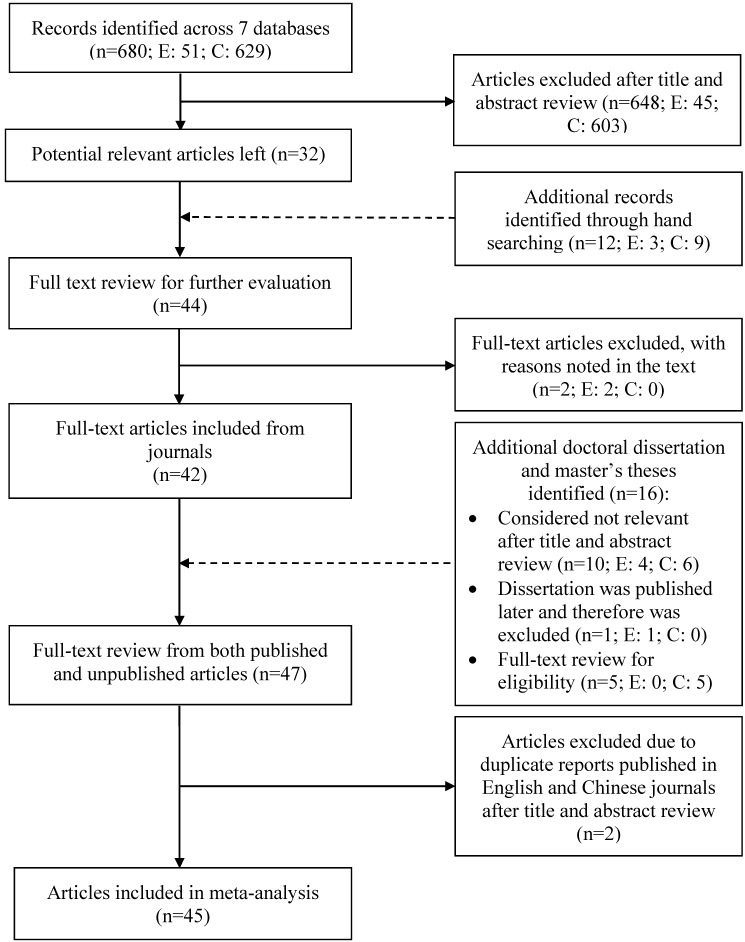
Selection process of included studies. E = English articles, and C = Chinese articles.

Finally, scrutiny of 47 included articles revealed two studies published in both English and Chinese journals had the same content. Therefore, two of the four articles were considered a duplicate and were removed. Finally, there were 45 articles included in this review. The study selection process is documented in the flow chart in Figure 1.

### 3.2. Study Characteristics

Overall, a total of 45 studies were selected, which consisted of 14,346 participants. The number of participants in each study ranged from 56 to 5961, the mean age from 51.59 to 72.35, and duration of disease from 5.9 years to 18 years. Both men and women were included in all selected studies. All studies were cross-sectional. As judged by Egger’s test, there was no evidence of publication bias being presented at 5% significance level for any of the factors except for self-efficacy and acceptance-resignation. The studies investigating the relationships between self-efficacy and DSM, and between self-efficacy and physical activity had publication bias (*p* = 0.014 and 0.013, respectively). The studies investigating the relationships between on acceptance-resignation and DSM also had publication bias (*p* = 0.039).

### 3.3. Factors Eligible for Meta-Analysis

Twenty-two factors eligible for meta-analysis were: four variables of coping strategy (confrontation, avoidance, acceptance-resignation, overall coping score), six variables of health beliefs (perceived susceptibility, perceived benefits, perceived severity, perceived barriers, and cues to action, overall health beliefs), three variables of locus of control (internality, powerful other externality, and chance externality), diabetes knowledge, depression, self-efficacy, social support, complication, female gender, health education, living alone, admission history.

**Figure 2:**
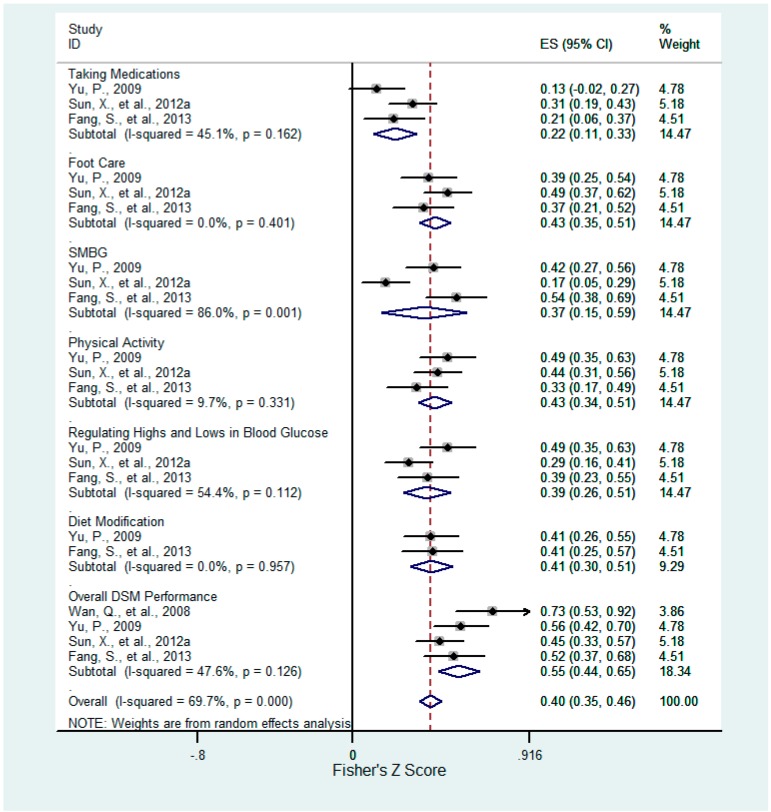
Forest plot for Fisher’s z-transformed correlation coefficient between confrontation and DSM practice.

**Figure 3:**
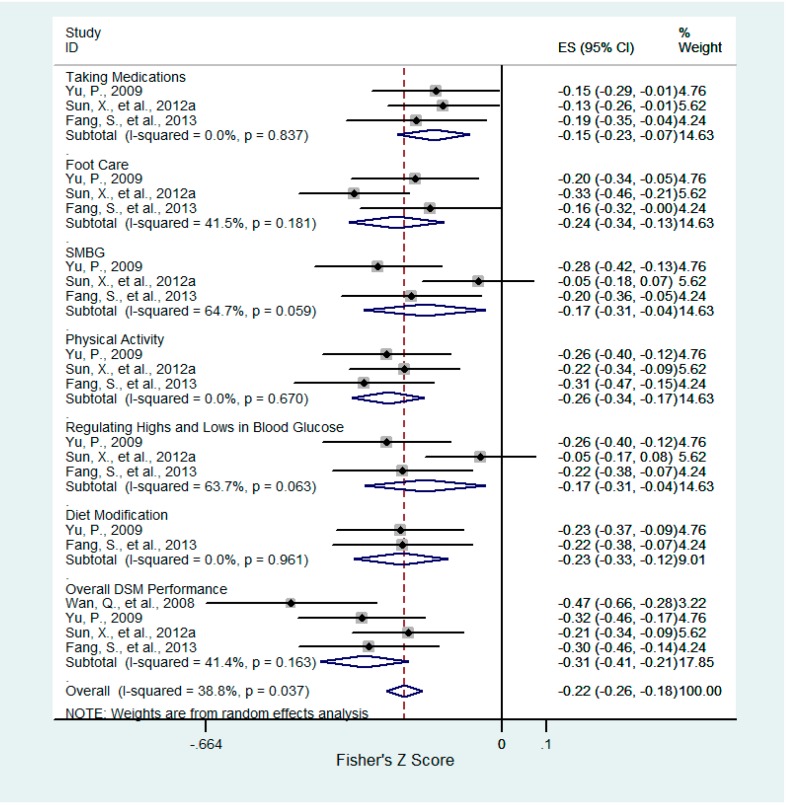
Forest plot for Fisher’s z-transformed correlation coefficient between acceptance-resignation and DSM practice.

**Figure 4:**
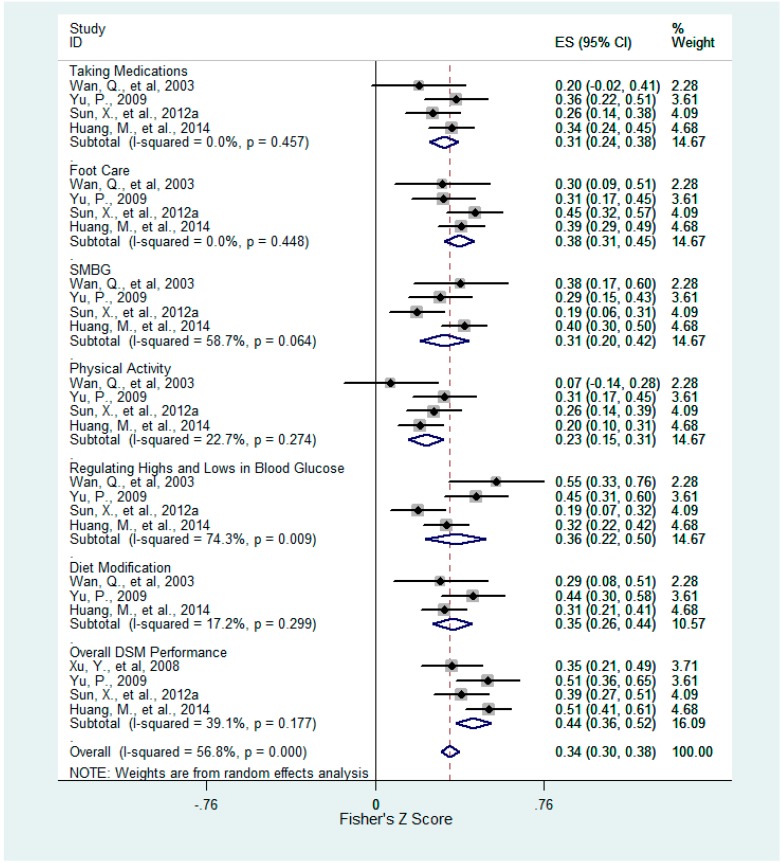
Forest plot for Fisher’s z-transformed correlation coefficient between health beliefs and DSM practice.

**Figure 5:**
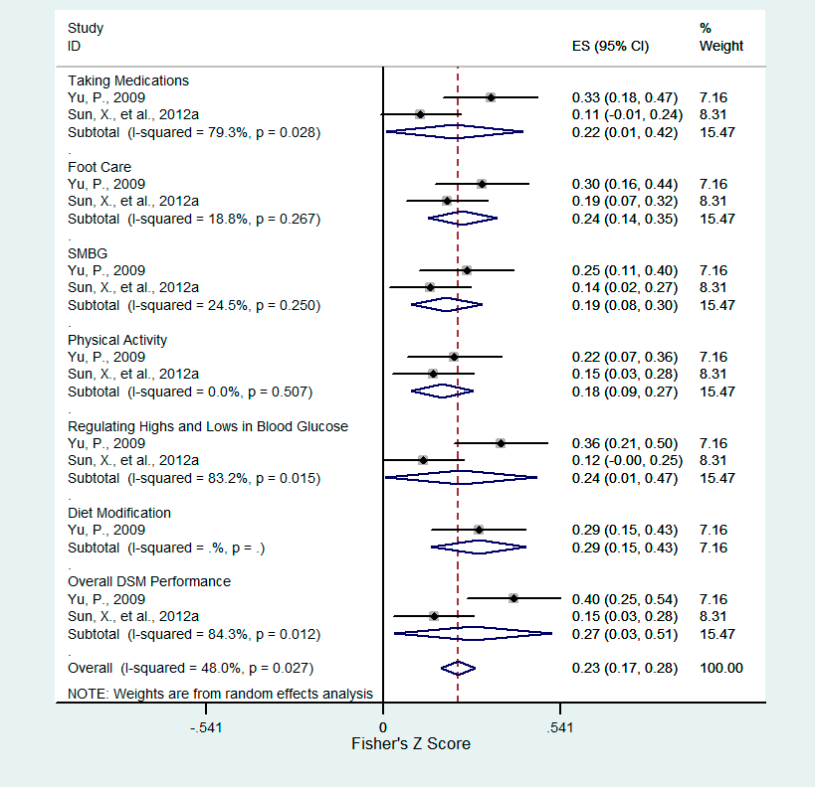
Forest plot for Fisher’s z-transformed correlation coefficient between perceived susceptibility and DSM practice.

**Figure 6:**
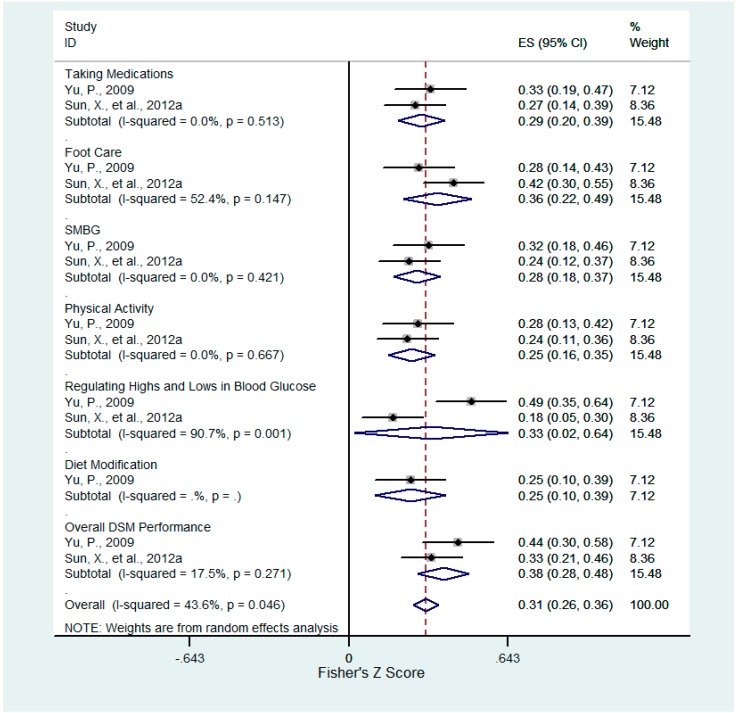
Forest plot for Fisher’s z-transformed correlation coefficient between perceived barriers and DSM practice.

**Figure 7:**
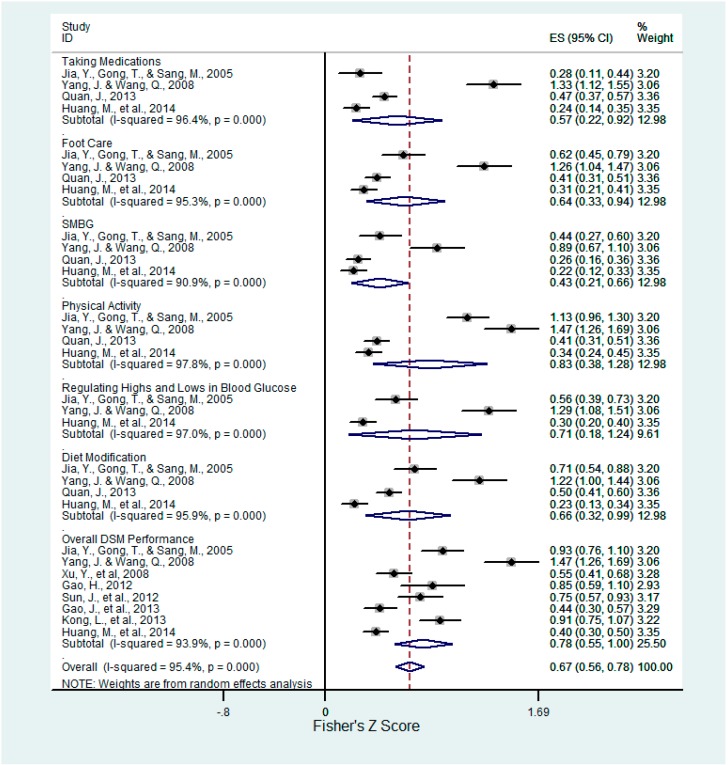
Forest plot for Fisher’s z-transformed correlation coefficient between self-efficacy and DSM practice.

Six of twenty-two factors were consistently associated with DSM and six aspects of DSM behaviors (Table 1). These factors included confrontation (Figure 2), acceptance-resignation (Figure 3), overall health beliefs (Figure 4), perceived susceptibility (Figure 5), perceived barriers (Figure 6), and self-efficacy (Figure 7). For each of the six aspects of DSM and the overall DSM performance, pooled correlation coefficients or beta coefficients were calculated based on different factors.

Although only a few factors showed substantial heterogeneity across studies (Table 2), random-effect models were used for better generalizability. The pooled effects of each factor on DSM behaviors is summarized in Table 1 and presented below.

**Table 1:** The Relationship between Diabetes Self-Management Behaviors and Twenty-Two Factors Eligible for Meta-Analysis.

Factors	Overall DSM Performance	Physical Activity	Taking Medications	SMBG	Foot Care	Regulating Highs and Lows in Blood Glucose	Diet Modification	Smoking
Overall Coping Score	0.33 (0.25, 0.41)	0.29 (0.21, 0.38)	NS	0.18 (0.09, 0.27)	0.30 (0.22, 0.39)	0.27 (0.18, 0.36)	0.22 (0.08, 0.35)	
Avoidance	0.11 (0.03, 0.19)	NS	NS	NS	0.13 (0.05, 0.21)	NS	NS	
Confrontation	0.50 (0.42, 0.57)	0.40 (0.33, 0.47)	0.22 (0.11, 0.32)	0.35 (0.15, 0.53)	0.40 (0.33, 0.47)	0.37 (0.26, 0.47)	0.39 (0.29, 0.47)	
Acceptance−Resignation	−0.30 (−0.39, −0.21)	−0.25 (−0.32, −0.17)	−0.15 (−0.23, −0.07)	−0.17 (−0.30, −0.04)	−0.23 (−0.33, −0.13)	−0.17 (−0.30, −0.04)	−0.22 (−0.32, −0.12)	
Overall Health Beliefs	0.42 (0.35, 0.48)	0.23 (0.15, 0.30)	0.30 (0.24, 0.36)	0.30 (0.20, 0.40)	0.36 (0.30, 0.42)	0.35 (0.22, 0.46)	0.34 (0.25, 0.41)	
Perceived Susceptibility	0.27 (0.03, 0.47)	0.18 (0.09, 0.27)	0.21 (0.01, 0.40)	0.19 (0.08, 0.29)	0.24 (0.14, 0.33)	0.23 (0.01, 0.44)	0.28 (0.15, 0.41)	
Perceived Benefits	0.41 (0.30, 0.51)	0.26 (0.17, 0.34)	0.31 (0.23, 0.39)	0.21 (0.08, 0.34)	0.37 (0.29, 0.45)	NS	0.37 (0.24, 0.49)	
Perceived Barriers	0.36 (0.27, 0.45)	0.25 (0.16, 0.33)	0.28 (0.20, 0.37)	0.27 (0.18, 0.35)	0.34 (0.22, 0.46)	0.32 (0.02, 0.57)	0.24 (0.10, 0.37)	
Perceived Severity	NS	NS	NS	NS	NS	NS	NS	
Cues to Action	0.26 (0.11, 0.41)	0.26 (0.17, 0.35)	0.20 (0.10, 0.28)	0.14 (0.04, 0.23)	0.20 (0.06, 0.33)	NS	0.41 (0.29, 0.52)	
Chance Externality	NS	NS	−0.13 (−0.22, −0.03)	NS	−0.08 (−0.15, −0.01)		NS	
Internality	0.15 (0.05, 0.24)	NS	NS	NS	NS		NS	
Powerful Others Externality	0.33 (0.24, 0.41)	NS	NS	0.23 (0.06, 0.37)	NS		NS	
Self−Efficacy	0.70 (0.56, 0.80)	0.68 (0.36, 0.86)	0.52 (0.22, 0.73)	0.41 (0.21, 0.58)	0.56 (0.31, 0.74)	0.61 (0.18, 0.84)	0.58 (0.31, 0.76)	
Social Support	0.45 (0.15, 0.67)	0.21 (0.14, 0.28)	NS	0.29 (0.11, 0.45)	0.27 (0.11, 0.41)	0.22 (0.13, 0.31)	NS	
Depressive Symptoms	−0.37 (−0.48, −0.25)	NS	NS	−0.28 (−0.40, −0.16)	−0.34 (−0.47, −0.19)	NS	−0.28 (−0.36, −0.19)	
Diabetes Knowledge	0.23 (0.08, 0.38)	NS	0.11 (0.04, 0.18)	0.19 (0.08, 0.30)	0.16 (0.08, 0.24)	0.25 (0.12, 0.38)	NS	−0.26 (−0.39, −0.13)
Complications *	NS							
Female *	1.89 (0.98, 2.79)							
Health Education *	NS	1.97 (0.11, 3.83)	NS	NS	2.14 (1.18, 3.10)	2.00 (0.05, 3.95)	NS	
Living Alone *	−2.74 (−4.01, 1.47)							
Admission History *	−1.53 (−6.90, 3.83)							

Notes: * beta values; NS, non-significant; 95% CI is given in parentheses.

**Table 2:** Study Heterogeneity Assessment.

Factors	Overall DSM Performance	Physical Activity	Taking Medication	SMBG	Foot Care	Regulating Highs and Lows in Blood Glucose	Diet Modification	Smoking
Overall Coping Score	NS	NS	*p* = 0.023 (80.6%)	NS	NS	NS		
Avoidance	NS	NS	NS	NS	NS	NS	NS	
Confrontation	NS	NS	NS	*p* = 0.001 (86.0%)	NS	NS	NS	
Acceptance-Resignation	NS	NS	NS	NS	NS	NS	NS	
Overall Health Beliefs	NS	NS	NS	NS	NS	*p* = 0.009 (74.3%)	NS	
Perceived Susceptibility	*p* = 0.012 (84.3%)	NS	*p* = 0.028 (79.3%)	NS	NS	*p* = 0.015 (83.2%)		
Perceived Benefits	NS	NS	NS	NS	NS	*p <* 0.001 (93.1%)		
Perceived Barriers	NS	NS	NS	NS	NS	*p* = 0.001 (90.7%)		
Perceived Severity	NS	NS	*p* = 0.014 (83.3%)	*p* = 0.020 (81.5%)	NS	*p* = 0.010 (85.1%)		
Cues to Action	NS	NS	NS	NS	NS	*p* = 0.007 (86.0%)		
Chance Externality		NS		NS	NS	NS	NS	
Internality		*p* = 0.015 (83.1%)		NS	*p* = 0.035 (77.6%)		NS	
Powerful Others Externality		*p <* 0.001 (95.0%)		*p* = 0.017 (82.3%)	*p* = 0.000 (93.7%)		*p <* 0.001 (90.3%)	
Self-Efficacy	*p <* 0.001 (95.3%)	*p <* 0.001 (97.8%)	*p <* 0.001 (96.4%)	*p <* 0.001 (90.9%)	*p* = 0.000 (95.3%)	*p <* 0.001 (97.0%)	*p <* 0.000 (95.9%)	
Social Support	*p <* 0.001 (95.4%)	NS	*p* = 0.001 (81.0%)	*p <* 0.001 (84.5%)	*p* = 0.002 (79.6%)		*p* = 0.000 (92.1%)	
Depressive Symptoms	NS	*p* = 0.001 (95.8%)	*p* = 0.002 (83.9%)	NS	NS	*p <* 0.001 (88.4%)	NS	
Diabetes Knowledge	*p* = 0.000 (82.7%)	NS	NS	NS	NS		*p <* 0.001 (98.1%)	
Complications	*p <* 0.001 (88.9%)							
Female Gender	NS							
Health Education	*p <* 0.001 (96.5%)	*p <* 0.001 (93.9%)	*p* = 0.002 (89.7%)	*p <* 0.001 (97.2%)	*p* = 0.030 (78.8%)	*p <* 0.001 (95.3%)	*p <* 0.001 (98.5%)	
Living Alone	NS							
Admission History	*p <* 0.001 (95.1%)							

Notes: I^2^ is given in parentheses; NS, non-significant.

#### 3.3.1. Coping Strategy

Four studies [11,24,25,26] investigated the association between some of the three forms of coping strategy and DSM practice. Significant associations were observed between a higher overall coping score and better DSM and every aspect of DSM behaviors except taking medication. Confrontation was positively associated with overall DSM performance and six aspects of DSM behavior, while acceptance-resignation was negatively associated with overall DSM performance and 6 aspects of DSM behavior. Although there was significant publication bias of studies on acceptance-resignation, the result of the Duval and Tweedie trim and fill method showed no changes. Avoidance was positively associated with overall DSM performance and foot care.

#### 3.3.2. Health Beliefs

Four studies [11,26,27,28] examined how health beliefs affect DSM. The pooled effects of overall health beliefs, perceived susceptibility and perceived barriers were significant on six aspects of DSM behaviors and DSM. Perceived benefits and cues to action were significantly related to better DSM behaviors except for regulating highs and lows in blood glucose. No significant association between perceived severity and DSM behavior was observed.

#### 3.3.3. Locus of Control

Two studies [29,30] examined the relationship between locus of control and DSM behaviors. Chance externality was negatively associated with taking medication and foot care. Internality was associated with overall DSM performance. Powerful others externality contributed to improvement in SMBG and overall DSM practice.

#### 3.3.4. Self-Efficacy

Eleven studies [19,23,24,28,30,31,32,33,34,35,36] reported relationship between self-efficacy and DSM behaviors. There was a consistent strong association between increased self-efficacy level and better DSM behaviors, with pooled estimates being significant on both seven specific aspects of DSM behaviors and overall DSM performance. Although there was publication bias regarding this factor and the overall DSM performance and physical activity, the results of the Duval and Tweedie trim and fill method showed that the effect size (ES) dropped from 0.78 to 0.55 for DSM (*p* < 0.001) and from 0.83 to 0.57 for physical activity (*p* = 0.019).

#### 3.3.5. Social Support

Six studies [19,23,27,28,30,37] investigated the association between social support and DSM. Social support was positively associated with overall DSM performance, engaging in physical activity, SMBG, foot care and regulating highs and lows in blood glucose.

#### 3.3.6. Depressive Symptoms

Three studies [38,39,40] examined the association between depressive symptoms and DSM behaviors. There was a consistent strong association of increased level of depressive symptoms being related to worse DSM behaviors, such as SMBG, foot care, diet modification, and overall DSM performance.

#### 3.3.7. Diabetes Knowledge

Seven studies [23,30,35,41,42,43,44] investigated the relationship between diabetes knowledge and DSM behaviors. Pooled results showed that diabetes knowledge was not only positively related to overall DSM practice, but also positively related to some specific DSM behaviors, such as taking medication, SMBG, foot care, regulating highs and lows in blood glucose, smoking cessation.

#### 3.3.8. Complications

Complications affected DSM behaviors differently across three observational studies [26,45,46]. There were conflicting results among the three studies. A negative association was reported in some studies [45,46], but not others [26]. However, no significant relationship was observed between complications and overall DSM performance.

#### 3.3.9. Female Gender

Two studies [26,47] examined the association between gender and DSM behaviors. After pooling the two studies’ results, female patients were found to have better overall DSM performance than their male counterparts.

#### 3.3.10. Health Education

Five studies [13,46,48,49,50] reported relationship between health education and DSM performance. The Diabetes Self-care Scale [51] was used in three studies [46,48,49], results were pooled to calculate the overall estimates. Pooled results showed physical activity, foot care, and regulating highs and lows in blood glucose were positively related to health education.

#### 3.3.11. Living Alone

Four studies [45,47,52,53] reported the association between living alone and DSM behaviors. The Diabetes Self-care Scale [51] was used in two studies [45,47], and results from these studies were pooled to calculate the overall estimates. Both studies reported that living alone was negatively associated with overall DSM performance, and the pooled estimates were significant.

#### 3.3.12. Admission History

Two studies [28,45] investigated the relationship between admission history and DSM practice. One study reported a negative association [28], but not the other [45]. There was no significant overall association between admission history and DSM practice.

### 3.4. Factors not Eligible for Meta-Analysis

These variables included age, duration of diabetes, educational level, household income level, provider-patient communication, health insurance coverage, chronic illness resources utilization (CIRU), marital status, and other factors.

#### 3.4.1. Age

It was not possible to pool the ES of age due to different categorizations of age groups and differing age ranges in the five studies [28,30,44,54,55]. There was inconsistent evidence of the relationship between age and DSM behaviors. One study found that diet modification, physical activity, SMBG, taking medication, and overall DSM were significantly lower among older adults (aged 60 or over) than middle-aged counterparts (aged between 40 and 59) [54]. In contrast, two other studies [28,55] reported a positive relationship between age and DSM.

#### 3.4.2. Duration of Diabetes

It was not possible to pool the ES of diabetes duration due to different categorizations among 12 studies [23,28,30,44,45,47,49,50,55,56,57,58]. Some studies found that patients with longer duration of disease managed diabetes better [23,45,49,50,55,56,57], but not others [28,58]. Longer duration of diabetes was positively related to SMBG, diet modification [30], and engaging exercise [25].

#### 3.4.3. Educational Level

It was not possible to pool the ES of educational level due to different categorizations among 6 studies [28,44,47,49,56,58,59]. Five studies reported a positive relationship between higher educational level and better DSM [28,49,56,58,59]. Patients with higher educational level were found to be more likely to maintain foot care [47], regulate highs and lows in blood glucose [27], follow diabetes nutritional recommendations, and SMBG [44].

#### 3.4.4. Household income Level

It was not possible to pool the ES of household income level due to different categorizations among three studies [26,49,58]. A significantly positive association was observed between higher household income level and better DSM in all three studies. Higher household income was also related to diet modification, taking medications, regulating highs and lows in blood glucose [58].

#### 3.4.5. Provider-Patient Communication

It was not possible to pool the ES of provider-patient communication due to heterogeneity [20,23]. Both studies reported that provider-patient communication was an independent, direct predictor of DSM [20,23].

#### 3.4.6. Health Insurance Coverage

It was not possible to pool the ES of health insurance coverage due to different categorizations among three studies. Those who had healthcare insurance tended to manage their diabetes better than those who did not have healthcare insurance [26]. Those who had healthcare insurance were also more likely to take medications [59], maintain foot care, SMBG, and engage in exercise [30].

#### 3.4.7. CIRU

It was not possible to pool the ES of CIRU due to heterogeneity [29,60]. Chronic illness support and resources may include community/neighborhood, family and friends, organizations, physician and healthcare team, media and policy, and personal support [61]. There was some limited evidence from two studies that greater CIRU was associated with better DSM practice [29,60].

#### 3.4.8. Marital Status

It was not possible to pool the ES of marital status due to heterogeneity [30,44]. Compared to unmarried individuals, married individuals were more likely to take medications, SMBG, and to engage in exercise [30,44].

## 4. Discussion

This is the first study to employ extensive literature search strategies to identify factors associated with DSM among Chinese adults with T2D. This study indicates that confrontation, resignation, overall health beliefs, perceived susceptibility, perceived barriers, and self-efficacy were factors consistently reported in the literature associated with DSM and six aspects of DSM behaviors. There is some limited evidence to suggest that provider-patient communication, married individuals, higher educational level, and higher household income level may also be linked to better DSM. Having healthcare insurance and utilizing chronic illness resources generally appeared to have a favorable effect on DSM. In addition, there were a number of factors for which the evidence is too limited to be able to ascertain its strength of association with DSM.

The Health Belief Model postulates that health behavior is determined by personal beliefs or perceptions about a disease and resources to reduce the occurrence [62]. Perceived susceptibility is one of the most important predictors for adopting health behavior [63], because when people believe that they are at risk for a disease, they will be more likely to do something to prevent it. In consistent with the postulation of the original Health Belief Model, perceived susceptibility was positively related to overall DSM and DSM behaviors. Perceived barriers are the most significant of all the constructs in determining health change [63]. In this study, perceived barriers were found to be positively related with overall DSM and DSM behaviors. However, the reasons for the positive relationship were not clear and warrant further investigations.

The experience of living with chronic disease can take a significant toll on the well-being of individuals in terms of emotional and physical discomforts. Living with chronic disease can become an important source of stress. Therefore, it is important to recognize the specific patterns of coping used by patients and to discern the effectiveness of their skills. Individuals with positive coping strategies such as confrontation tend to be more proactive in learning to manage their disease. In contrast, individuals with negative coping strategies such as avoidance or acceptance-resignation may not be willing to follow management recommendations. Therefore, diabetes educators should focus on helping patients develop positive coping strategies.

The three forms of locus of control were found to be associated with DSM. In line with previous studies [64,65], internal locus control was associated with better DSM. According to Rodin, individuals with internal locus of control may adhere more closely to prescribed regimen because they believe disease can be controlled via personal ability and actions. In contrast, individuals with external locus of control may not closely follow treatment regimen because they believe that disease is determined largely by chance or other persons [66]. However, our results partly conflict with the latter point of view. In China, patients with T2D rely heavily on family and friends for decision-making around their treatment much more than patients in western cultures which value individuality and independent decision-making [23,30]. Therefore, powerful others may become an important source of support for some specific DSM behaviors..

In Chinese culture, strong family bonds and family intimacy are important and highly valued [67]. A cohesive and supportive family may provide patients with an opportunity to express feelings and concerns [23]. When DSM is viewed as shared responsibility with entire family, patients may adopt DSM behaviors more easily and feel more confident in managing diabetes [68]. Therefore, it has been suggested that family members be involved in interventions to promote DSM [23], and family-focused interventions may be more effective in improving DSM performance than individual-focused interventions. This is especially important for those who live alone. Without family support, DSM practice may become a big challenge. Other support alternatives, such as health care providers and community, may be crucial to facilitating DSM practice in this patient population.

Special considerations should be given to patients with depressive symptoms. Mental disorder is generally viewed as degrading not only to the patient, but also to the entire family in Chinese culture [69]. Patients may not actively seek treatment, which could adversely limit their ability perform effective DSM. Depression screening therefore should be integrated into DSM assessment using validated depressing screening tools such as Center for Epidemiologic Studies Depression Scale [70]. Patients with severe depressive symptoms may be referred to a psychiatrist for further treatment. Framing the referral as common may remove some of existing stigma concerning depression and its treatment. However, lifestyle intervention that includes healthy diet and regular exercise have been proven effective for depressed individuals with diabetes [71], and it may be sufficient for patients who are mildly or moderately depressed. Unfortunately, no guidance is currently available on when to refer people who are identified as having depression, and it should become a focus of active research in the future.

The systematic review and meta-analysis has some limitations. The quality assessment checklist was adapted from previously published assessment checklists [16,17], which has not been rigorously tested. In addition, although extensive and diverse search strategies were used to locate all possibly available literature, some grey literature, such as conference proceedings, was still difficult to find. Furthermore, the patients in current study were middle-aged and older Chinese adults. The findings of the study may not be generalized to young adults with T2D. Finally, The Chinese version of the Health Belief Model Scale was developed by Chen, and the author did not give detailed explanation of overall health beliefs. It is suggested that the five subscales of the Health Belief Model Scale should be analyzed individually [72]. So we did not discuss the relationship between overall health beliefs and DSM in details.

## 5. Conclusions

In conclusion, this is the first study to quantitatively synthesize the data on factors associated with DSM behaviors in Chinese adults with T2D. This study has several implications for clinical practice and future research. First of all, clinicians can develop effective strategies to improve certain DSM behaviors that patients are less likely to perform, such as SMBG or foot care. Secondly, identifying factors that influence DSM is the first step in developing theory-based interventions to promote short- and long-term health outcomes. Future research should focus on developing and testing a conceptual model that can be used to enhance DSM practice. Finally, to contribute to long-term reduction in diabetes-related mortality, researchers should examine ways to extend and maintain DSM behaviors among this population.

## Supplementary Files

Supplementary File 1
