# Supplementary material for: Factors Influencing Self-Management in Chinese Adults with Type 2 Diabetes: A Systematic Review and Meta-Analysis
Source: Int J Environ Res Public Health. 2015 Sep 10;12(9):11304–27. doi: 10.3390/ijerph120911304 (PMC4586677; doi:10.3390/ijerph120911304)
Supplement: Supplementary File 1 [file ijerph-12-11304-s001.pdf]

Factors Influencing Self-Management in Chinese Adults with Type 2 Diabetes: A Systematic Review and Meta-Analysis

---

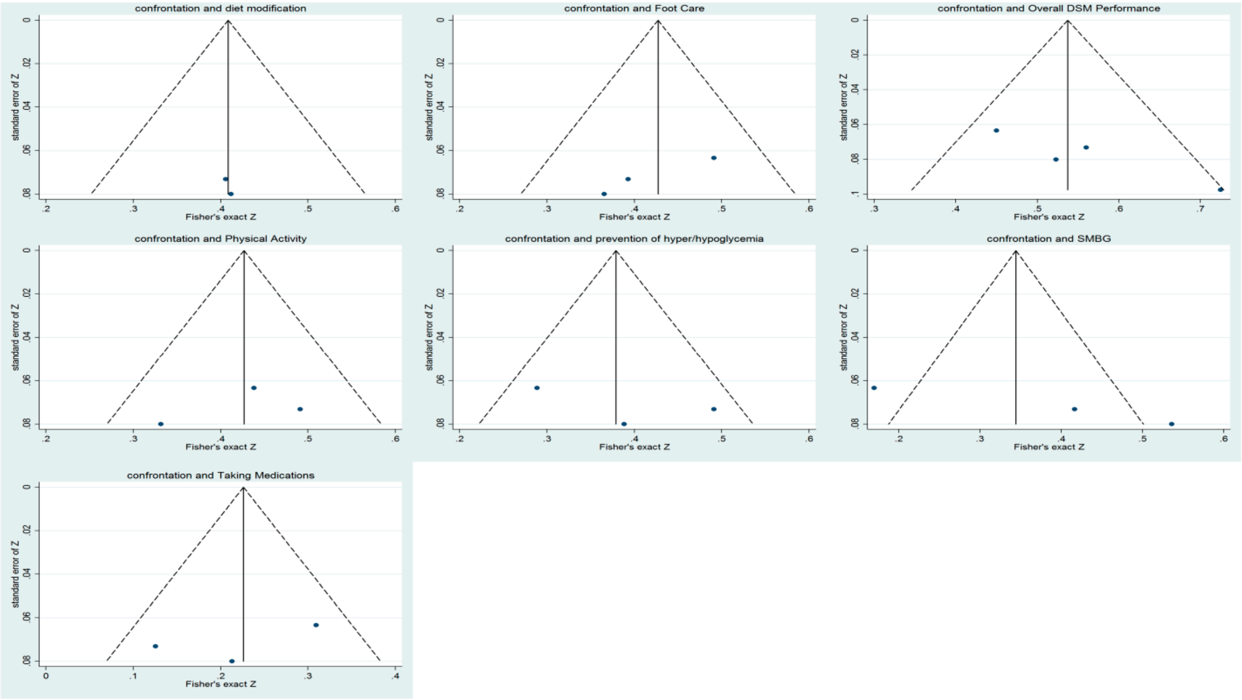

Figure S1. Funnel plot for confrontation and DSM behaviors.

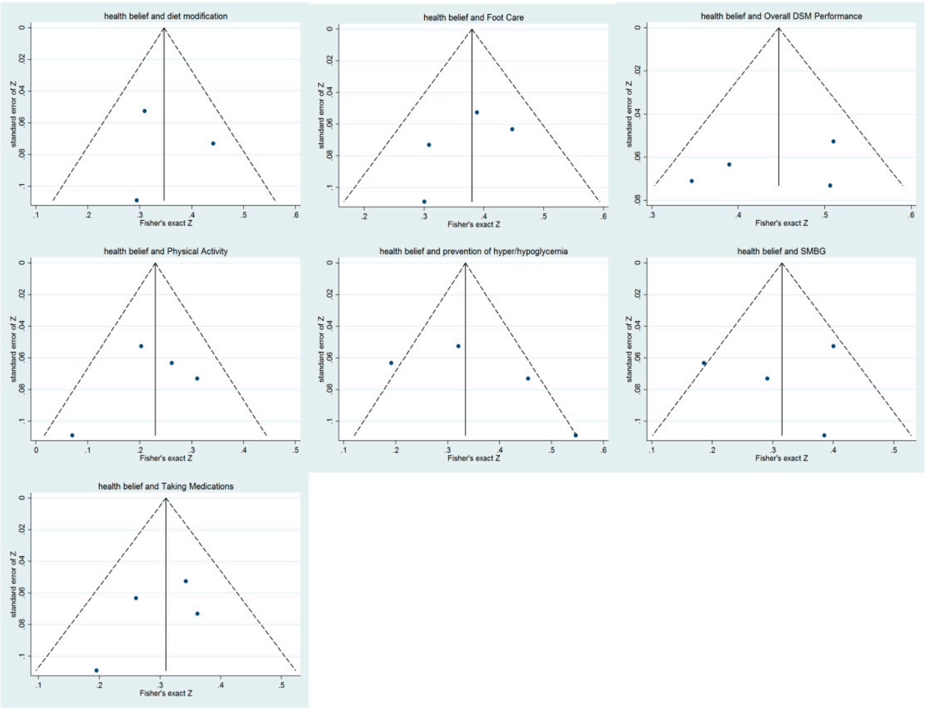

Figure S2. Funnel plot for health beliefs and DSM behaviors.

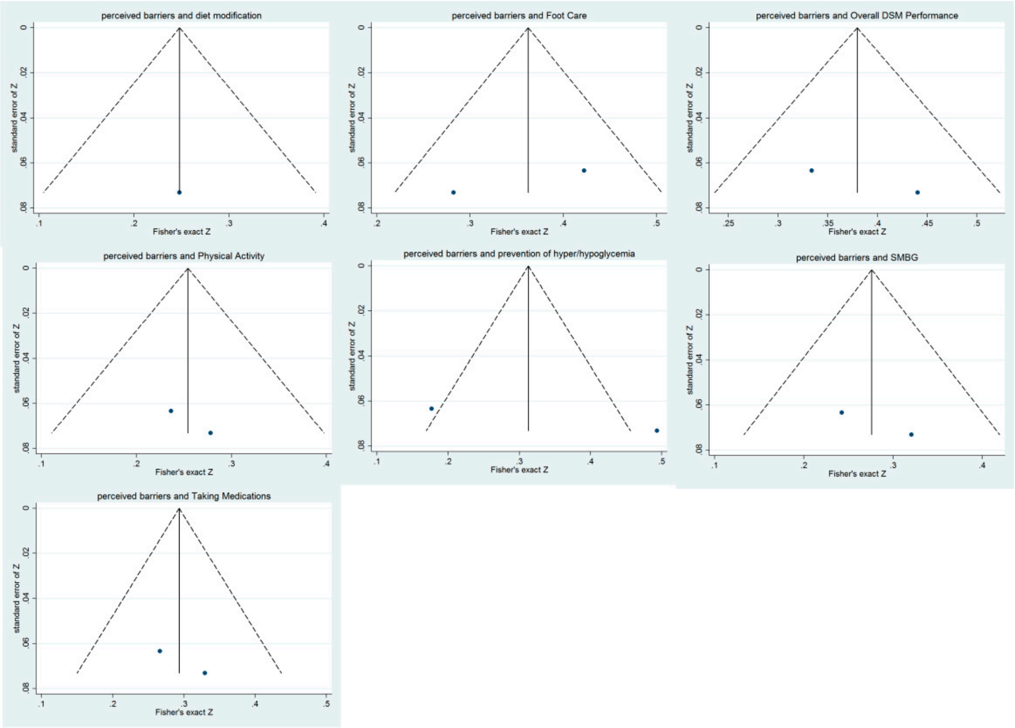

Figure S3. Funnel plot for perceived barriers and DSM behaviors.

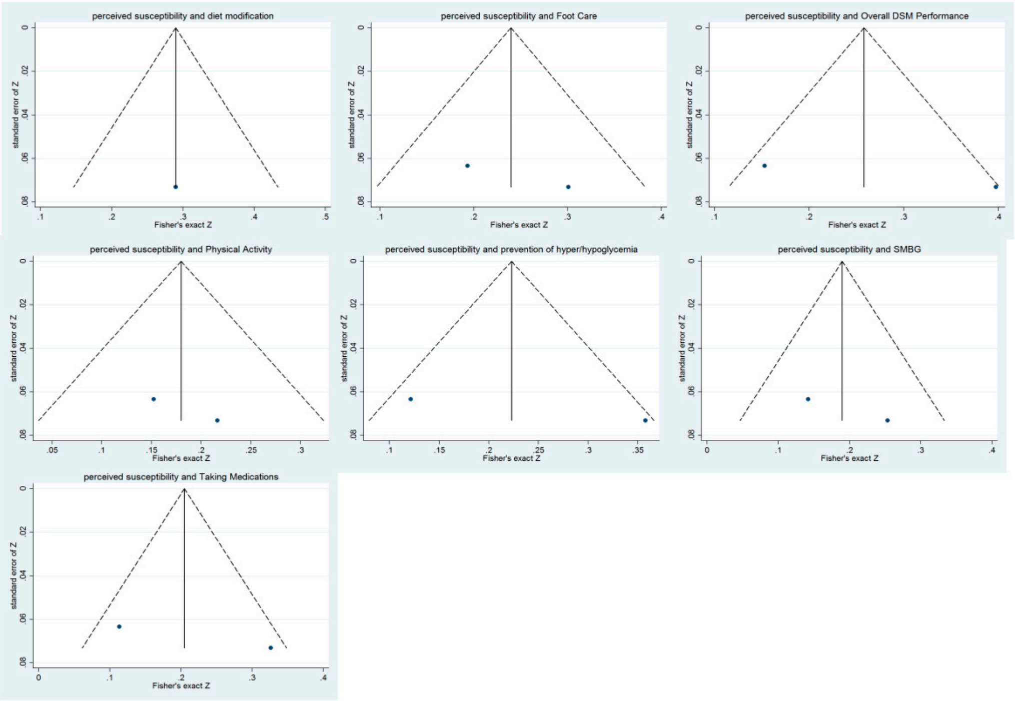

Figure S4. Funnel plot for perceived susceptibility and DSM behaviors.

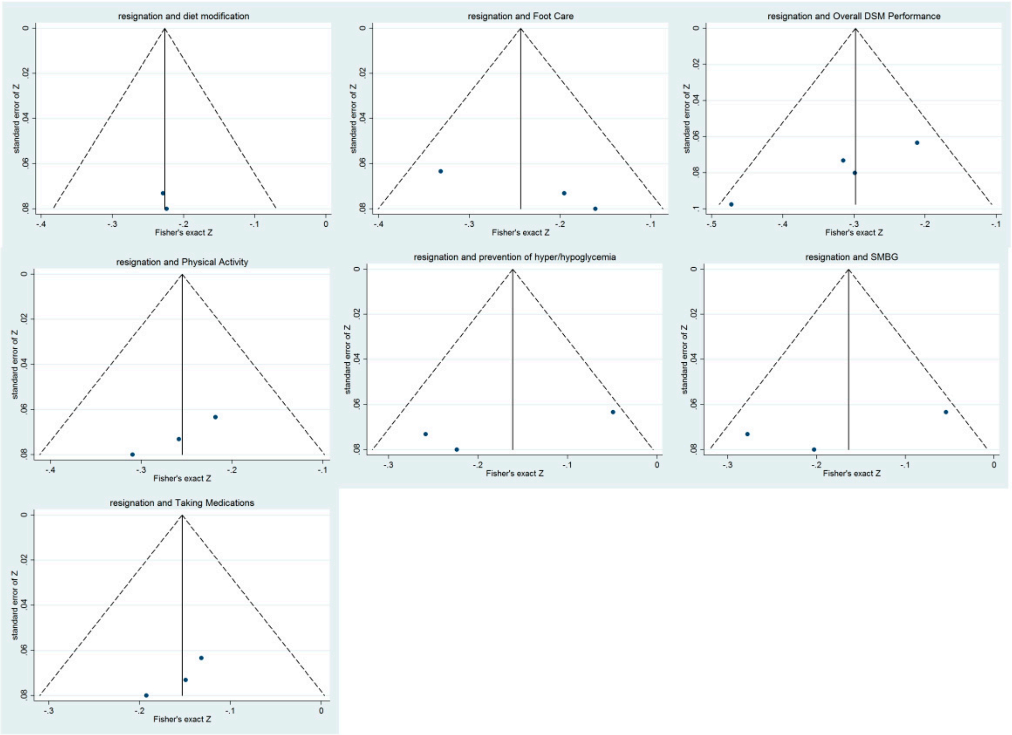

Figure S5. Funnel plot for acceptance-resignation and DSM behaviors.

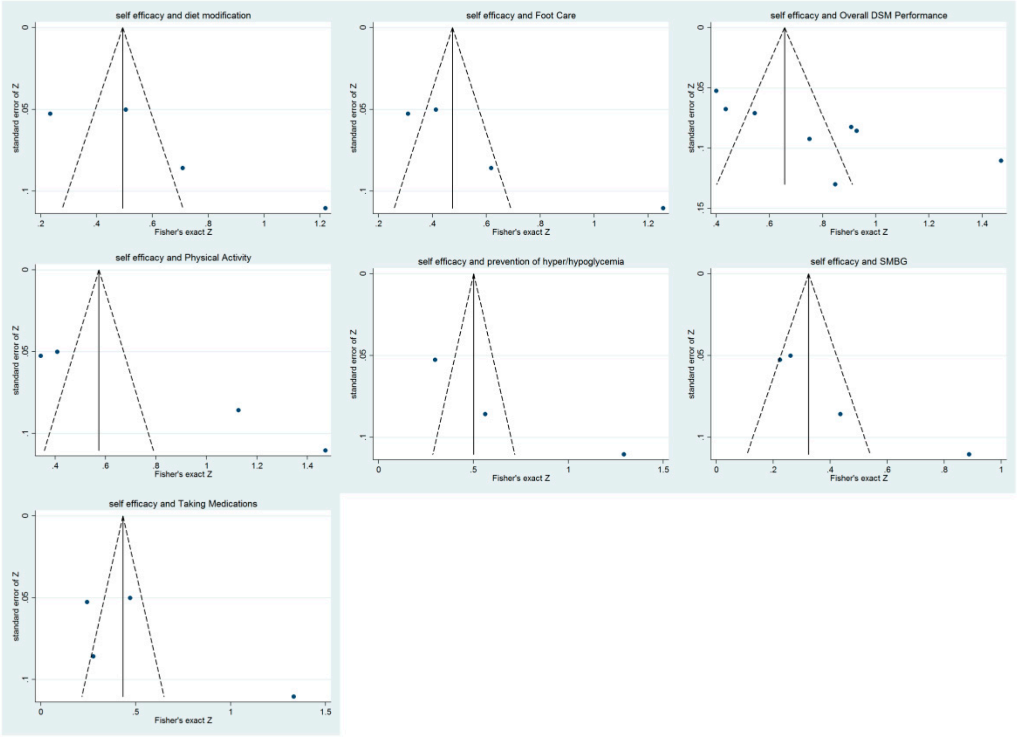

Figure S6. Funnel plot for self-efficacy and DSM behaviors.

Table S1. PICOS review questions.

Population	Chinese Adults Aged 18 Years or Older with Type 2 Diabetes
Intervention	None
Comparison	None
Outcome	DSM behaviors, related factors to DSM behaviors
Study Design	Case-control, cross-sectional, and cohort studies

Table S2. PubMed search terms.

#1.	Diabetes mellitus, type 2 [MeSH Terms] OR ketosis-resistant diabetes [Text Word] OR maturity onset diabetes [Text Word] OR non-insulin dependent diabetes [Text Word] OR type 2 diabetes [Text Word] OR stable diabetes [Text Word] OR diabetes mellitus, type II [text word] OR maturity onset diabetes mellitus [Text Word] OR MODY [Text Word] OR NIDDM [Text Word] OR adult onset diabetes mellitus [Text Word] OR diabetes mellitus noninsulin dependent [Text Word]
#2.	Self care [MeSH Terms] OR self-care [Text Word] OR self management [Text Word] OR self-management [Text Word]
#3.	Adults [MeSH Terms] OR adults [Text Word] AND Chinese [Text Word]
#4.	Chinese patients [Text Word] OR China [Text Word]
#5.	#3 OR #4
#6.	Case-control study [Publication Type] OR cross-sectional study [Publication Type] OR cohort study [Publication Type]
#7.	English [Language] OR Chinese [Language]
#8.	#1 AND #2 AND #5 AND #6 AND #7

Table S3. Quality appraisal results of included studies.

	**Study Question**		**Study Population**				**Outcome Measurement**				**Statistical Analysis**			**Funding**
**Criteria Articles**	Statement of study purpose given	Title consistent with purpose of study	Sample size pre-determined prior to study initiation	Adequate number of subjects enrolled	Inclusion criteria clearly described	Eligible subjects who refused to enroll were described	Reliable (Cronbach's alpha ≥.70) measure of DSM	DSM behaviors defined in the study	Reliable (Cronbach's alpha ≥.70) measure of related factors	Related factors defined in the study	Descriptive measures identified for all important variables	Text clearly understandable	Reported statistical tests appear to be clearly identified	Sources of support for study specified
Cong, J., et al., 2011	Y	Y	N	U	Y	N	Y	Y	Y	Y	Y	Y	Y	N
Du, L. et al., 2012	Y	Y	N	U	Y	N	Y	Y	U	Y	Y	Y	Y	N
Fang, S., et al., 2013	Y	Y	N	U	Y	N	Y	Y	P	P	Y	Y	Y	N
Gao, H. 2012	Y	Y	N	U	Y	N	U	Y	U	Y	Y	Y	Y	N
Gao, J., et al., 2013	Y	Y	N	U	Y	Y	Y	Y	N	P	Y	Y	Y	Y
Guo, X., et al., 2012	Y	Y	N	U	Y	N	Y	Y	N	Y	Y	Y	Y	Y
Hu, J., et al., 2012	Y	Y	N	U	Y	Y	Y	Y	Y	Y	Y	Y	Y	Y

Table S3. *Cont.*

	Study Question		Study Population				Outcome Measurement				Statistical Analysis			Funding
**Criteria Articles**	Statement of study purpose given	Title consistent with purpose of study	Sample size pre-determined prior to study initiation	Adequate number of subjects enrolled	Inclusion criteria clearly described	Eligible subjects who refused to enroll were described	Reliable (Cronbach's alpha ≥.70) measure of DSM	DSM behaviors defined in the study	Reliable (Cronbach's alpha ≥.70) measure of related factors	Related factors defined in the study	Descriptive measures identified for all important variables	Text clearly understandable	Reported statistical tests appear to be clearly identified	Sources of support for study specified
Hua, L., 2010	Y	Y	N	U	N	N	Y	Y	Y	Y	Y	Y	Y	N
Huang, J., et al., 2013	Y	Y	N	U	Y	N	Y	Y	Y	Y	Y	Y	Y	Y
Huang, M., et al., 2014	Y	Y	N	U	Y	N	Y	Y	Y	Y	Y	Y	Y	Y
Jia, Y., Gong, T., Sang, M., 2005	Y	Y	N	U	Y	N	Y	Y	Y	Y	Y	Y	Y	N
Jia, Y., Wang, J., & Liu, W., 2005	Y	Y	N	U	Y	N	Y	Y	Y	Y	Y	Y	Y	N
Jia, Y. et al., 2004	Y	Y	N	U	N	N	U	Y	Y	N	Y	Y	Y	N

Table S3. *Cont.*

	Study Question		Study Population				Outcome Measurement				Statistical Analysis			Funding
**Criteria Articles**	Statement of study purpose given	Title consistent with purpose of study	Sample size pre-determined prior to study initiation	Adequate number of subjects enrolled	Inclusion criteria clearly described	Eligible subjects who refused to enroll were described	Reliable (Cronbach's alpha ≥.70) measure of DSM	DSM behaviors defined in the study	Reliable (Cronbach's alpha ≥.70) measure of related factors	Related factors defined in the study	Descriptive measures identified for all important variables	Text clearly understandable	Reported statistical tests appear to be clearly identified	Sources of support for study specified
Kong, L., et al., 2013	Y	N	N	U	Y	N	Y	Y	Y	P	Y	Y	Y	N
Kong, Y. & Chen, Y., 2009	Y	Y	N	U	Y	N	U	Y	Y	Y	Y	Y	Y	N
Liang, Y., et al., 2009	Y	Y	N	U	N	N	U	Y	Y	Y	Y	Y	Y	N
Lin, T., et al., 2009	Y	Y	N	U	N	N	Y	Y	P	N	Y	Y	Y	N
Lin, T., et al., 2008	Y	Y	N	U	N	N	Y	Y	Y	Y	Y	Y	Y	N
Ma, J. & Mo, G., 2010	Y	Y	N	U	Y	N	U	Y	U	Y	Y	Y	Y	Y
Meng, F., 2010	Y	Y	Y	Y	Y	N	Y	Y	Y	Y	Y	Y	Y	N

Table S3. *Cont.*

	Study Question		Study Population				Outcome Measurement				Statistical Analysis			Funding
**Criteria Articles**	Statement of study purpose given	Title consistent with purpose of study	Sample size pre-determined prior to study initiation	Adequate number of subjects enrolled	Inclusion criteria clearly described	Eligible subjects who refused to enroll were described	Reliable (Cronbach's alpha ≥.70) measure of DSM	DSM behaviors defined in the study	Reliable (Cronbach's alpha ≥.70) measure of related factors	Related factors defined in the study	Descriptive measures identified for all important variables	Text clearly understandable	Reported statistical tests appear to be clearly identified	Sources of support for study specified
Mu, Y., 2008	Y	Y	Y	Y	Y	N	Y	Y	Y	Y	Y	Y	Y	N
Quan, J., 2013	Y	Y	Y	Y	Y	N	Y	Y	Y	Y	Y	Y	Y	N
Shi, L., et al., 2010	Y	Y	N	U	Y	N	Y	Y	Y	N	Y	Y	Y	N
Sun, J., et al., 2012	Y	Y	N	U	Y	N	Y	Y	Y	Y	Y	Y	Y	N
Sun, X., et al., 2012a	Y	P	N	U	Y	N	Y	Y	P	Y	Y	Y	Y	N
Sun, X., et al., 2012b	Y	Y	N	U	Y	N	Y	Y	P	N	Y	Y	Y	N
Wan, Q., et al., 2010	Y	Y	N	U	Y	N	Y	Y	Y	Y	Y	Y	Y	Y
Wan, Q. et al., 2003	Y	Y	N	U	Y	N	Y	Y	P	Y	Y	Y	Y	N

Table S3. *Cont.*

	Study Question		Study Population				Outcome Measurement				Statistical Analysis			Funding
**Criteria Articles**	Statement of study purpose given	Title consistent with purpose of study	Sample size pre-determined prior to study initiation	Adequate number of subjects enrolled	Inclusion criteria clearly described	Eligible subjects who refused to enroll were described	Reliable (Cronbach's alpha ≥.70) measure of DSM	DSM behaviors defined in the study	Reliable (Cronbach's alpha ≥.70) measure of related factors	Related factors defined in the study	Descriptive measures identified for all important variables	Text clearly understandable	Reported statistical tests appear to be clearly identified	Sources of support for study specified
Wan, Q., et al., 2008	Y	Y	N	U	Y	N	Y	Y	U	Y	Y	Y	Y	N
Wang, J. Q. & Tak-Ying Shiu, A. 2004	Y	Y	N	U	Y	N	Y	N	Y	N	Y	Y	Y	N
Wang, J. & Zhang, X., 2002	Y	Y	N	U	Y	N	Y	Y	Y	Y	Y	Y	Y	N
Wang, L. & Zhang, J., 2012	Y	Y	N	U	Y	N	Y	Y	Y	Y	Y	Y	Y	N

Table S3. *Cont.*

	**Study Question**		**Study Population**				**Outcome Measurement**				**Statistical Analysis**			**Funding**
**Criteria Articles**	Statement of study purpose given	Title consistent with purpose of study	Sample size pre-determined prior to study initiation	Adequate number of subjects enrolled	Inclusion criteria clearly described	Eligible subjects who refused to enroll were described	Reliable (Cronbach's alpha ≥.70) measure of DSM	DSM behaviors defined in the study	Reliable (Cronbach's alpha ≥.70) measure of related factors	Related factors defined in the study	Descriptive measures identified for all important variables	Text clearly understandable	Reported statistical tests appear to be clearly identified	Sources of support for study specified
Wang, Q., Wan, Q., & Shang, S., 2009	Y	Y	N	U	Y	N	Y	Y	U	N	Y	Y	Y	N
Wang, X., Lan, W., et al., 2012	Y	Y	N	U	Y	N	U	Y	Y	Y	Y	Y	Y	N
Wang, X., Lv, W., et al., 2013	Y	Y	N	U	Y	N	Y	Y	Y	Y	Y	Y	Y	Y
Wang, X., Zhang, J., et al., 2012	Y	Y	N	U	Y	N	Y	Y	P	Y	Y	Y	Y	N
Wang, Y., et al., 2013	Y	Y	N	U	Y	N	N	N	Y	Y	Y	Y	Y	Y

Table S3. *Cont.*

	Study Question		Study Population				Outcome Measurement				Statistical Analysis			Funding
**Criteria Articles**	Statement of study purpose given	Title consistent with purpose of study	Sample size pre-determined prior to study initiation	Adequate number of subjects enrolled	Inclusion criteria clearly described	Eligible subjects who refused to enroll were described	Reliable (Cronbach's alpha ≥.70) measure of DSM	DSM behaviors defined in the study	Reliable (Cronbach's alpha ≥.70) measure of related factors	Related factors defined in the study	Descriptive measures identified for all important variables	Text clearly understandable	Reported statistical tests appear to be clearly identified	Sources of support for study specified
Xia, Z. & Yang, T., 2011	Y	Y	N	U	N	N	U	Y	Y	Y	Y	Y	Y	N
Xu, Y., et al., 2008	Y	Y	Y	Y	Y	N	N	Y	P	N	Y	Y	Y	N
Yang, J. & Wang, Q., 2008	Y	Y	N	U	N	N	Y	Y	Y	N	Y	Y	Y	
Yu, P., 2009	Y	Y	N	U	Y	N	Y	Y	P	Y	Y	Y	Y	N
Yu, Z., et al., 2012	Y	Y	N	U	N	N	U	Y	P	Y	Y	Y	Y	N
Zhao, W. 2007	Y	Y	N	U	Y	N	U	N	U	N	Y	Y	Y	N
Zhong, X., et al., 2011	Y	Y	Y	Y	Y	N/A	Y	Y	Y	N	Y	Y	Y	N
Zhu, F., et al., 2011	Y	Y	N	U	N	N	Y	Y	Y	Y	Y	Y	Y	Y

Note: Y = yes; N = no; U = unclear; P = partial; N/A = not available.

© 2015 by the authors; licensee MDPI, Basel, Switzerland. This article is an open access article distributed under the terms and conditions of the Creative Commons Attribution license (http://creativecommons.org/licenses/by/4.0/).
